# Application of cotton straw biochar and compound *Bacillus* biofertilizer decrease the bioavailability of soil cd through impacting soil bacteria

**DOI:** 10.1186/s12866-022-02445-w

**Published:** 2022-01-26

**Authors:** Yongqi Zhu, Xin Lv, Jianghui Song, Weidi Li, Haijiang Wang

**Affiliations:** Agricultural College, Shihezi University, 832003 Shihezi, Xinjiang P. R. China

**Keywords:** Bacteria diversity, Biochar, Biofertilizer, Cd bioavailability, Enzyme activity

## Abstract

**Background:**

Cd seriously threatens soil environment, remedying Cd in farmland and clearing the response of soil environment to modifiers in Cd-contaminated soils is necessary. In this study, the effects of cotton straw biochar and compound *Bacillus* biofertilizer used as modifiers on the biochemical properties, enzyme activity, and microbial diversity in Cd-contaminated soils (1, 2, and 4 mg·kg^−1^) were investigated.

**Results:**

The results showed that both cotton straw biochar and compound *Bacillus* biofertilizer could improve the soil chemical characteristics, including the increase of soil C/N ratio, electrical conductance (EC) and pH, and the most important decrease of soil available Cd content by 60.24% and 74.34%, respectively (*P < 0.05*). On the other hand, adding cotton straw biochar and compound *Bacillus* biofertilizer in Cd stressed soil also improved soil biological characteristics. Among them, cotton straw biochar mainly through increasing soil alkaline phosphatase activity and improve bacteria abundance, compound *Bacillus* biofertilizer by increasing soil invertase, alkaline phosphatase, catalase, and urease activity increased bacterial community diversity. On the whole, the decrease of soil available Cd was mainly caused by the increase of soil pH, C/N, urease and alkaline phosphatase activities, and the relative abundance of *Acidobacteria* and *Proteobacteria*.

**Conclusions:**

In summary, the applications of cotton straw biochar and compound *Bacillus* biofertilizer could decrease soil available Cd concentration, increase soil bacterial community diversity and functions metabolism, and reduce the damage of Cd stress, compared with cotton straw biochar, compound *Bacillus* biofertilizer was more effective in immobilizing Cd and improving soil environmental quality.

## Background

Cd contamination caused by sewage irrigation and application of fertilizers and pesticides is very common in the farmlands in China [1–3]. Higher Cd accumulation negatively impacts soil biochemical properties [4–6] and microbial activity [7]. However, soil enzymes play important role in various biochemical processes. When the soil is contaminated by Cd, soil urease, phosphatase, and catalase activities are obviously decreased [8]. For example, Wang et al. [9] have shown that the phosphatase activity in Cd-contaminated soil (10 mg·kg^−1^) could be obviously decreased, while no difference could be found in the urease activity. Moreover, soil microbes, an essential part of the ecosystem, are also greatly impacted by Cd contamination [10]. Fritze [11] has shown that the number of *Actinomycetes* and fungi could be decreased in Cd-treated soil. Cd mainly accumulates in the surface soil. Higher Cd accumulation always decreases the activities of microbes through damaging the cell membranes and DNA structure and influencing cell functions [12–14], and causes toxicity to microbes. Therefore, for Cd-contaminated soils, it is indispensable to find an eco-friendly remediation method to improve the degraded soil ecosystem.

Biotic and abiotic remediation are important methods for the remediation of heavy metal-contaminated soils [15]. Abiotic remediation includes electro kinetic remediation, soil replacement, soil isolation, chemical leaching, organic matter fixation, etc. [15]. Biochar is an environmentally friendly adsorbent that could be used for abiotic remediation, with the characteristics of low cost and high efficiency [16]. It could reduce soil available Cd concentration [17], and increase soil pH [16, 18], organic carbon concentration [19], enzyme activity [20], and biochemical properties. Bioremediation uses microorganisms or plants to detoxify heavy metals or remove from soils. Compound *Bacillus* biofertilizer, an atoxic multifunctional fertilizer, could be used in the inoculation with functional bacteria to enhance soil fertility and quality, and reduce heavy metal toxicity [21]. Previous study has reported that the Cd-removal rate after inoculating with *Bacillus* in soil reached more than 80.01%, and the adsorption capacity was 62.0 - 159.5 mg Cd [22]. Moreover, the application of modifiers is certain to impact soil microbes and enzyme activity. Chen et al. [23] have shown that the application of biochar (40 t ha^−1^) could increase phosphatase and catalase activities, and change the microbial biomass by changing soil carbon and nitrogen. In the remediation of Pb- and As-contaminated soils using biochar, the relative abundance of *Actinomycetes* could be increased obviously, while the relative abundances of *Acidbacteria* and *Chloroflexi* were decreased [24]; however, the urease activity could be increased obviously after application of compound *Bacillus* biofertilizer [25]. The planting area of cotton in China is as high as 3339.2 kha in 2019, accounting for 9.98% of the total area of cotton fields in the world. The planting area of cotton in Xinjiang Province is up to 2540.5 kha, accounting for 70.03% of the total area in China. In recent years, due to the rapid development of industry and unreasonable field management, such as excessive application of chemical fertilizer, pesticides and plastic film residues, the potential ecological risk of soil Cd contamination in farmlands increases rapidly in Xinjiang, China [26, 27].

At present, the researches on the remediation of Cd contaminated soil are mostly based on acidic soil, while there are few reports based on alkaline soil in arid and semi-arid areas. Besides, biochar and biofertilizer are commonly used in soil remediation, but the mechanisms of the remediation of Cd contaminated alkaline soil by the application of biochar and biofertilizer are still not clear. Therefore, in this study, cotton straw biochar and compound *Bacillus* biofertilizer were selected as modifiers to explore their effects on the diversity of bacterial communities in Cd-contaminated alkaline soils, and clarify the key bacteria involved in the remediation. We hypothesized that: (1) There may be differences in the biochemical characteristics and microbial diversity of alkaline soil contaminated by different concentrations of Cd; and (2) Applications of cotton straw biochar and compound *Bacillus* biofertilizer may change soil enzyme activity and bacterial diversity and have different effects on the key bacterial communities in the soil.

## Results

### Soil biochemical properties

The applications of cotton straw biochar and compound *Bacillus* biofertilizer had different effects on soil biochemical properties (Table 1). After the application of Cd, the soil C/N ratios in the H1T, H2T, and H3T treatments decreased by 3.91%, 7.31%, and 14.55%, respectively, while the soil EC increased by 90.38%, 61.54%, and 28.85%, respectively (*P < 0.05*), compared with those in the control group (H0T treatment).

**Table 1:** Effect of the applications of biochar and biofertilizer on soil biochemical properties

Cd (mg·kg^-1^)	Modifiers(%)	pH	C/N ratio	EC (ms·cm^-1^)
H0	T	7.44±0.21 b	10.54±0.39 d	2.08±0.02 h
	B	8.49±0.24 a	16.93±0.78 bc	2.88±0.02 ef
	J	8.42±0.24 a	18.51±0.53 a	2.41±0.02 g
H1	T	7.23±0.20 b	9.60±0.38 d	3.96±0.03 b
	B	8.58±0.25 a	16.41±0.62 c	4.91±0.04 a
	J	8.57±0.24 a	17.62±0.48 abc	4.96±0.04 a
H2	T	7.97±0.23 ab	10.55±0.36 d	3.36±0.03 c
	B	8.63±0.24 a	16.47±0.54 c	4.88±0.04 a
	J	8.45±0.24 a	18.66±0.48 a	4.77±0.04 a
H3	T	7.56±0.21 b	9.57±0.33 d	2.68±0.02 gf
	B	8.46±0.21 a	16.92±0.51 bc	3.30±0.03 cd
	J	8.57±0.24 a	17.98±0.40 ab	3.03±0.05 de
Regression Analysis				
H		ns	ns	ns
BJ		ns	*	ns
BJ*H		ns	*	ns

T, no modifiers; B, 3% biochar was applied; J, 1.5% biofertilizer was applied; H0, no Cd; H1, 1 mg·kg^−1^ of Cd was applied; H2, 2 mg·kg^−1^ of Cd was applied; H3, 4 mg·kg^−1^ of Cd was applied. Different lowercase letters in the same column indicate significant differences (*P < 0.05*). **, *P < 0.01*; *, *0.01 < P < 0.05*; ns, *P ≥ 0.05*

The soil pH, C/N ratio, and EC could be increased after the applications of cotton straw biochar and compound *Bacillus* biofertilizer (Table 1). The soil C/N ratio and EC in the cotton straw biochar and compound *Bacillus* biofertilizer treatments were higher than those in the control group. For example, soil pH, C/N ratio, and EC in the H2B treatment increased by 10.78%, 56.11%, and 45.24%, respectively, and those in the H2J treatment increased by 6.02%, 76.87%, and 41.96%, respectively, compared with those in the H2T treatment (*P < 0.05*).

Regression analysis showed that the application of Cd had no effect on soil pH, C/N ratio, and EC (*P > 0.05*), and the application of modifiers greatly impacted soil C/N ratio (*P < 0.05*). The modifiers and Cd greatly impacted soil C/N ratio (*P < 0.05*), but no differences were found in soil pH and EC (*P > 0.05*).

### Effects of cotton straw biochar and compound *Bacillus* biofertilizer on soil available Cd

**Fig. 1:**
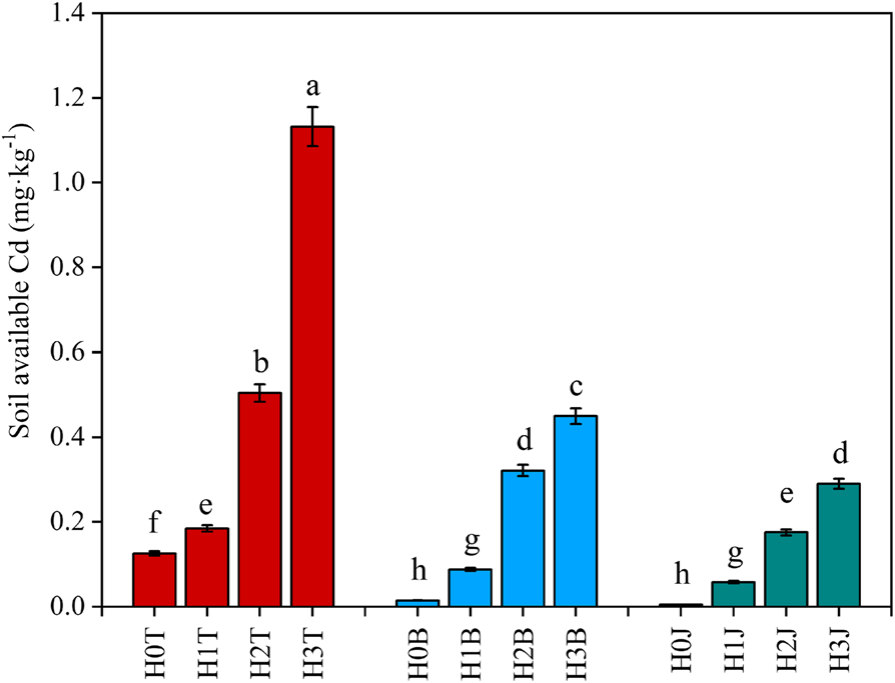
Effect of the applications of cotton straw biochar and compound *Bacillus* biofertilizer on soil available Cd. Values show the mean of five replicates ± SE. Means followed by same small letters are not significant different at *P < 0.05* by using the Duncan test

The soil available Cd concentration in the H1T, H2T, and H3T treatments increased after the application of exogenous Cd (*P < 0.05*) (Fig. 1). The highest soil available Cd concentration was 1.13 mg·kg^−1^ which was found in the H3T treatment. The soil available Cd concentration decreased in the cotton straw biochar (H1B, H2B, and H3B) and compound *Bacillus* biofertilizer (H1J, H2J, and H3J) treatments (*P < 0.05*). Soil available Cd concentration in the H0B and H0J treatments decreased by 88.26% and 95.96%, respectively (*P < 0.05*), compared with that in the H0T treatment. Soil available Cd concentration in the H1B and H1J treatments decreased by 52.32% and 68.54%, respectively (*P < 0.05*), compared with that in the H1T treatment. Soil available Cd concentration in the H2B and H2J treatments decreased by 36.30% and 65.17%, respectively (*P < 0.05*), compared with that in the H2T treatment. Soil available Cd concentration in the H3B and H3J treatments decreased by 60.24% and 74.34%, respectively (*P < 0.05)*, compared with that in the H3T treatment.

### Effects of modifiers and Cd on soil enzyme activities

**Fig. 2:**
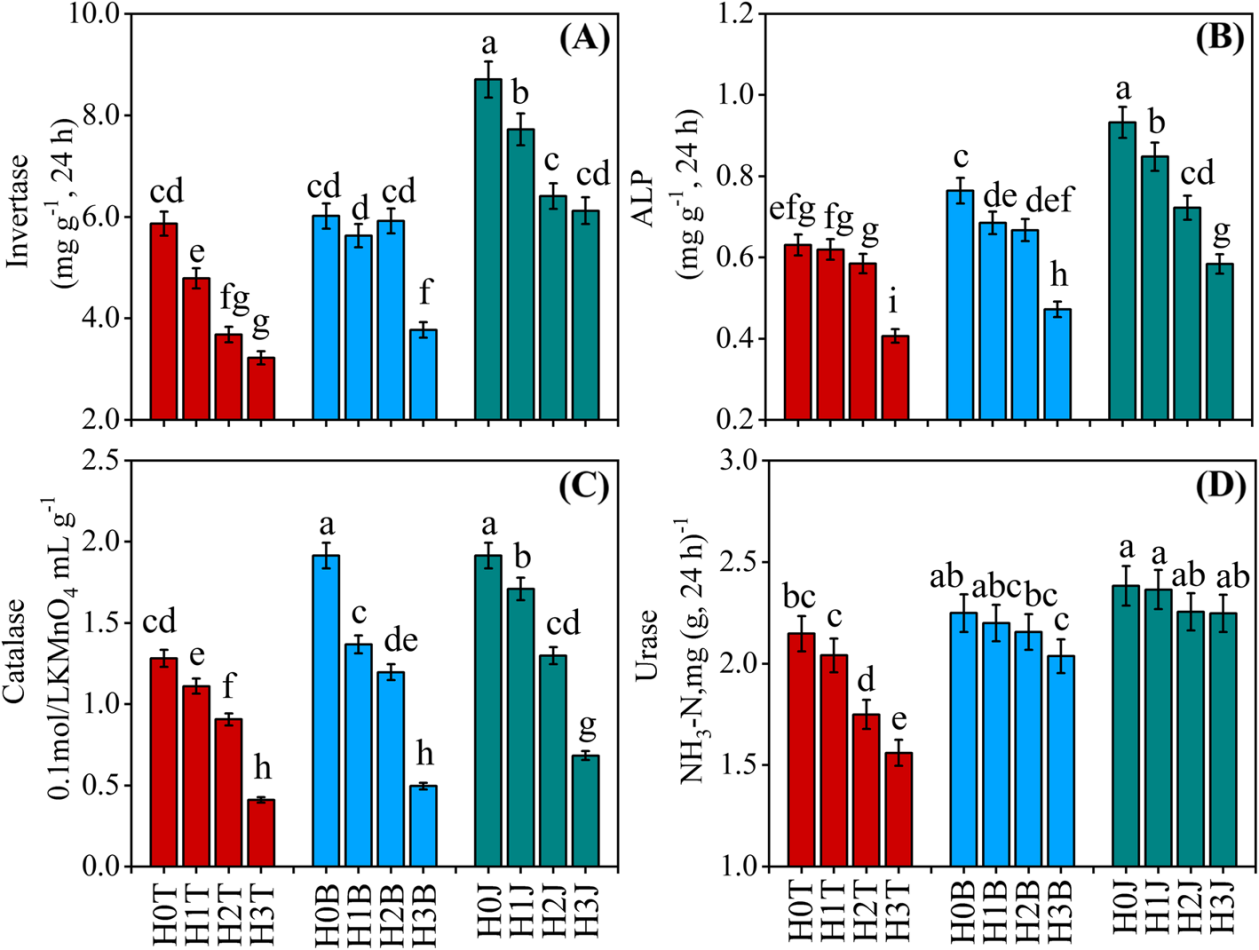
Effect of the applications of cotton straw biochar and compound *Bacillus* biofertilizer on soil invertase (**A**), urease (**B**), alkaline phosphatase (**C**), and catalase (**D**) activities. Values show the mean of five replicates ± SE. Means followed by same small letters are not significant different at *P < 0.05* by using the Duncan test

Soil enzyme activity decreased after the application of exogenous Cd (Fig. 2). Soil invertase activity in the H1T, H2T, and H3T treatments decreased by 18.36%, 37.25%, and 45.07, respectively (*P < 0.05*), compared with that in the H0T treatment. Soil alkaline phosphatase activity (ALP) in the H2T and H3T treatments decreased by 7.21% and 35.53%, respectively (*P < 0.05*), and soil urease activity decreased by 18.54% and 27.33%, respectively (*P < 0.05*), compared with those in the H0T treatment. The activities of soil invertase, alkaline phosphatase, catalase, and urease in the H3T treatment were the lowest, which decreased by 45.07%, 35.53%, 68.01%, and 27.33%, respectively (*P < 0.05*), compared with those in the H0T treatment.

Soil invertase enzyme activity increased after the applications of cotton straw biochar and compound *Bacillus* biofertilizer (Fig. 2 A). Soil invertase activity in the H1B and H1J treatments increased by 17.51% and 61.29%, respectively, compared with that in the H1T treatment (*P < 0.05*). The activity of alkaline phosphatase also increased after the applications of cotton straw biochar and compound *Bacillus* biofertilizer, and difference was found between cotton straw biochar and compound *Bacillus* biofertilizer treatments (*P < 0.05*). For example, soil alkaline phosphatase activity in the H3B and H3J treatments increased by 16.16% and 43.74%, respectively (*P < 0.05*), compared with that in the H3T treatment (Fig. 2B). Soil catalase activity in the H1B and H1J treatments increased by 23.08% and 53.85%, respectively (*P < 0.05*), compared with that in the H1T treatment (Fig. 2 C). Soil urease activity in the H2B and H2J treatments increased by 13.27% and 28.94%, respectively (*P < 0.05*), compared with that in the H2T treatment (Fig. 2D).

### Effects of the applications of cotton straw biochar and compound *Bacillus* biofertilizer on soil microbial community diversity

Coverage indices showed that the sequencing coverage indices of each sample was more than 97.97%, which could reflect the reliability of this sequencing result (Table 2). The Simpson index increased after the applications of cotton straw biochar and compound *Bacillus* biofertilizer (*P < 0.05*). The Simpson’s diversity index in the H2B and H2J treatments increased by 66.67% and 50.88%, respectively (*P < 0.05*), compared with that in the H2T treatment. The Chao1 index in the cotton straw biochar and compound *Bacillus* biofertilizer treatments increased. The Chao1 index in the H0B and H0J treatments increased by 20.21% and 17.66%, respectively (*P < 0.05*), compared with that in the H0T treatment; similar trends were also found in the H3B and H3J treatments.

**Table 2:** Changes in microbial diversity after the applications of biochar and biofertilizer

Cd (mg·kg^−1^)	Modifiers (%)	Diversity index of soil microbial community		
		Simpson	Chao1	Coverage
H0	T	0.0070±0.0002 f	1801±51.99 e	0.9853±0.028 a
	B	0.0117±0.0003 c	2165±62.48 bcd	0.9819±0.028 a
	J	0.0095±0.0003 d	2119±61.17 cd	0.9820±0.028 a
H1	T	0.0059±0.0002 g	1964±56.69 de	0.9835±0.028 a
	B	0.0070±0.0002 f	2030±58.61 d	0.9841±0.028 a
	J	0.0128±0.0004 b	2397±69.22 a	0.9809±0.028 a
H2	T	0.0057±0.0002 g	2083±60.13 cd	0.9824±0.028 a
	B	0.0095±0.0005 d	2453±70.81 a	0.9800±0.028 a
	J	0.0086±0.0004 de	2277±65.74 abc	0.9799±0.028 a
H3	T	0.0078±0.0004 ef	2333±67.36 ab	0.9797±0.028 a
	B	0.0090±0.0003 d	2251±64.99 abc	0.9811±0.028 a
	J	0.0315±0.0009 a	2251±64.98 abc	0.9806±0.028 a
Regression Analysis				
H		ns	*	**
BJ		ns	*	**
BJ*H		ns	*	**

T, no application of modifiers; B, 3% biochar was applied; J, 1.5% biofertilizer was applied; H0, no application of Cd; H1, 1 mg·kg^−1^ of Cd was applied; H2, 2 mg·kg^−1^ of Cd was applied; H3, 4 mg·kg^−1^ of Cd was applied. Different lowercase letters in the same column indicate significant differences (*P < 0.05*) in Cd content. **, *P < 0.01;* *, *0.01 < P < 0.05*; ns, *P ≥ 0.05*

Regression analysis showed that Cd and modifiers greatly impacted Chao1 index and Coverage index (*P < 0.05*), but there was no difference in the Simpson’s diversity index (*P > 0.05*). Moreover, the applications of Cd and modifiers had a combined effect on soil microbial diversity (*P < 0.05*).

#### Effect of the applications of cotton straw biochar and compound *Bacillus* biofertilizer on the relative abundance of soil bacteria

**Fig. 3:**
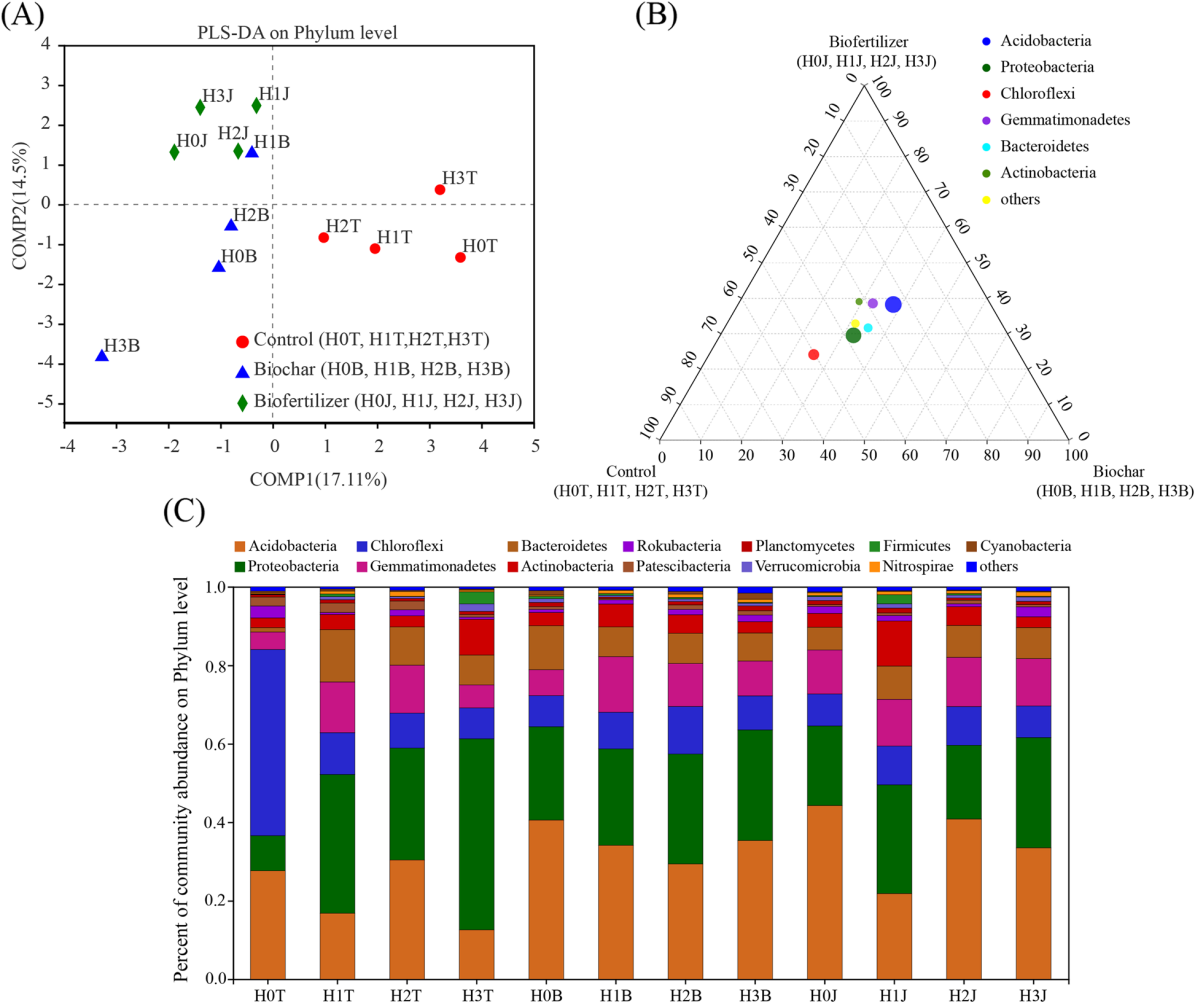
PLS-DA analysis (**A**), ternary phase diagram analysis (**B**) and relative abundances of soil microbes (**C**) after the applications of cotton straw biochar and compound *Bacillus* biofertilizer at phylum level

**Fig. 4:**
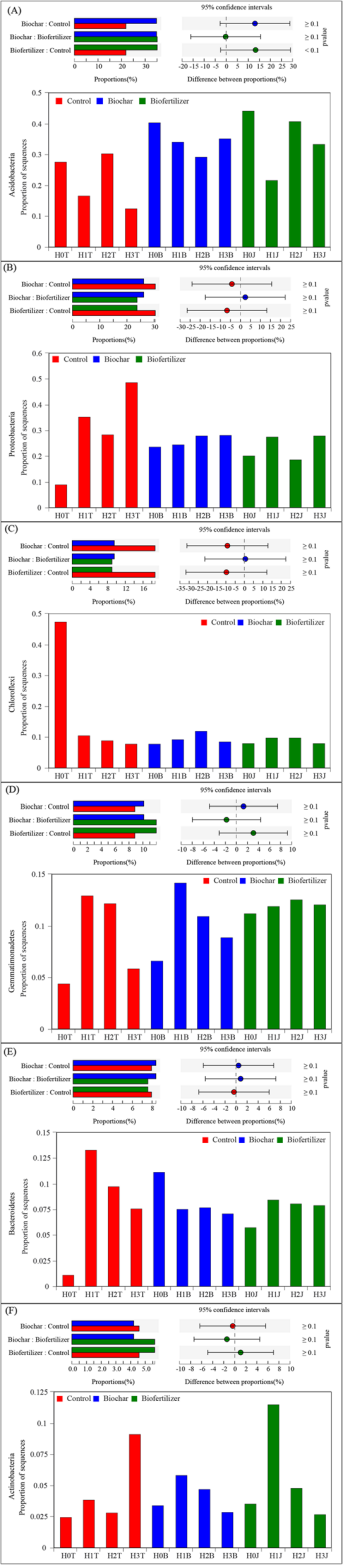
Analysis of significant differences between groups of dominant phyla in different treatments. Abbreviations: The relative abundances of *Acidobacteria* (**A**), *Proteobacteria* (**B**), *Chloroflexi* (**C**), *Gemmatimonadetes* (**D**), *Bacteroidetes* (**E**), and *Actinobacteria* (**F**) under the comparison among biochar treatments (H0B, H1B, H2B, and H3B), biofertilizer treatments (H0J, H1J, H2J, and H3J), and control (H0T, H1T, H2T, and H3T)

According to the PLS-DA analysis, it can be seen that the composition of soil bacterial community in Cd pollution treatment (Control) and modifier (cotton straw biochar and compound *Bacillus* biofertilizer) is significantly different on the COMP1 axis. There were significant differences between cotton straw biochar and compound *Bacillus* biofertilizer treatments on the COMP2 axis (Fig. 3 A). The applications of modifiers and Cd could obviously impact the relative abundance of bacteria. Among them, *Acidobacteria*, *Proteobacteria*, *Chloroflexi*, *Gemmatimonadetes*, *Bacteroidetes*, and *Actinobacteria* were the dominant phyla, accounting for 91.27-95.52% of bacteria in soil samples (Fig. 3 C). Ternary phase diagram analysis showed that the composition and distribution ratio of phylum level were different in different samples. *Chloroflexi* was abundant in the control treatments (H0T, H1T, H2T, H3T). *Acidobacteria* is more abundant in the cotton straw biochar treatments (H0B, H1B, H2B, H3B) and compound *Bacillus* biofertilizer treatments (H0J, H1J, H2J, H3J) (Fig. 3B). For the control treatments (H0T, H1T, H2T, H3T), cotton straw biochar treatments (H0B, H1B, H2B, H3B), and compound *Bacillus* biofertilizer treatments (H0J, H1J, H2J, H3J) samples were tested for significant difference between groups, the results showed that compared with the control treatments (H0T, H1T, H2T, H3T), *Acidobacteria*, *Gemmatimonadetes* and *Bacteroidetes* increased in the cotton straw biochar treatments (H0B, H1B, H2B, H3B) (*P ≥ 0.1*), *Proteobacteria*, *Chloroflexi*, *Actinobacteria* decreased (*P ≥ 0.1*); Compared with the control treatments (H0T, H1T, H2T, H3T), *Acidobacteria*, *Gemmatimonadetes*, and *Actinobacteria* increase (*P ≥ 0.1*) in the compound *Bacillus* biofertilizer treatments (H0J, H1J, H2J, H3J). *Proteobacteria*, *Chloroflexi* and *Bacteroidetes* decreased (*P ≥ 0.1*) (Fig. 4).

The relative abundance of *Acidobacteria* in the H1T and H3T treatments decreased by 10.77% and 14.92%, respectively, and the relative abundance of *Chloroflexi* decreased by 36.94% and 39.64%, respectively, compared with those in the H0T treatment. However, the relative abundances of *Acidobacteria* and *Gemmatimonadetes* in the H0B treatment increased by 12.63% and 2.09%, respectively, compared with those in the H0T treatment. Similar trends were found in the relative abundances of *Acidobacteria* and *Gemmatimonadetes* in the H3B treatment. The relative abundances of *Acidobacteria* and *Proteobacteria* in the H0B treatment increased by 16.88% and 11.58%, respectively, and the relative abundances of *Acidobacteria* and *Gemmatimonadetes* in the H2J treatment increased by 10.48% and 0.39%, respectively, compared with those in the H2T treatment. The relative abundances of *Acidobacteria* and *Gemmatimonadetes* in the H3J treatment also increased by 20.83% and 6.12%, respectively, compared with that in the H3T treatment (Fig. 3 C).

##### Cotton straw biochar and compound *Bacillus* biofertilizer modulate soil bacterial metabolic functions in Cd-stressed soil

**Fig. 5:**
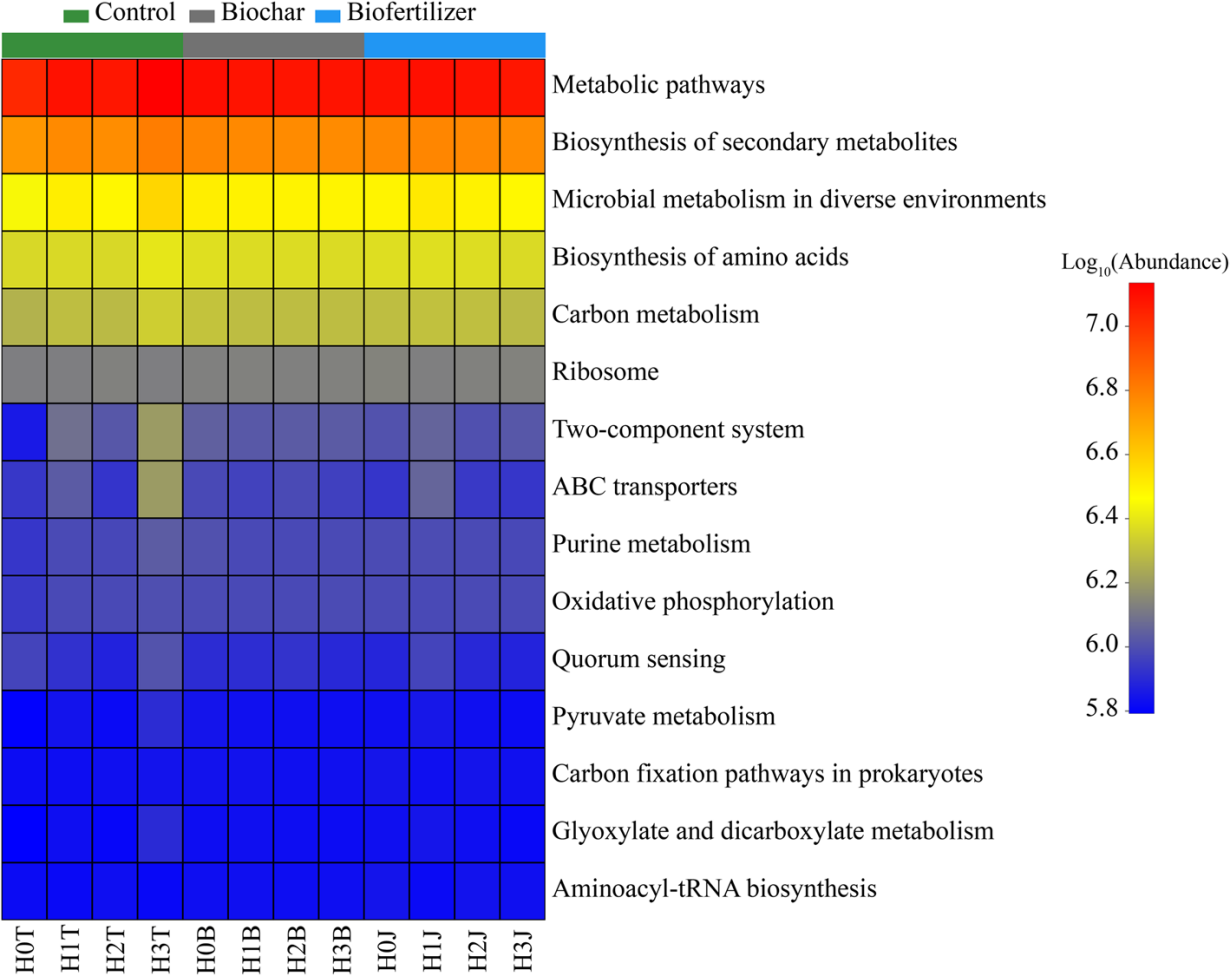
Effects of cotton straw biochar and compound *Bacillus* biofertilizer on the relative abundance of predicted bacterial metabolic functions

In addition to the changes of soil bacterial community, the metabolic functions of soil bacteria can also be used to evaluate the improvement of soil Cd pollution. Cotton straw biochar and compound *Bacillus* biofertilizer treatment increased the abundance values of most bacterial metabolic functions (Fig. 5). Compared with the H0T treatment, H0B and H0J treatments increased the bacteria metabolic function of top 15. Compared with the H1T treatment, H1B and H1J treatments increased the biosynthesis of amino acids, ribosome, aminoacyl-tRNA biosynthesis. Compared with the H2T treatment, H2B and H2J treatments increased the microbial metabolism in diverse environments, biosynthesis of amino acids, carbon metabolism, ABC transporters, quorum sensing, pyruvate metabolism, carbon fixation pathways in prokaryotes, glyoxylate and dicarboxylate metabolism. Compared with the H3T treatment, H3B and H3J treatments increased the ribosome. Among them, the improvement effect of compound *Bacillus* biofertilizer on bacterial metabolic function was better than that of cotton straw biochar under different concentrations of Cd pollution.

### Relationship between soil microbial diversity and biochemical properties

**Fig. 6:**
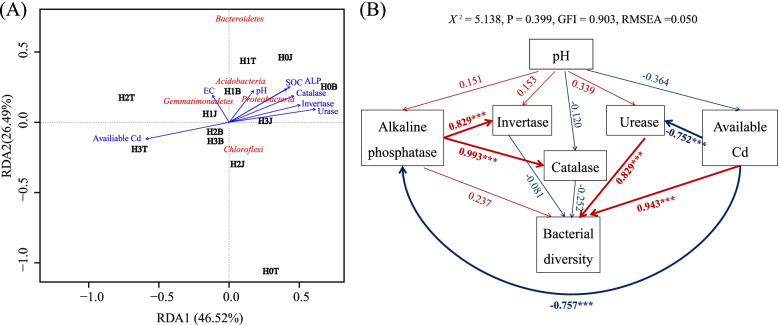
RDA analysis (**A**) and structural equation modeling (SEM) (**B**) between soil biochemical properties and soil microbial structure at phylum level. Abbreviations: soil available Cd, available Cd; pH, soil pH; Urase, soil urase activity; Sucrase, soil sucrase activity; Catalase, soil catalase activity; ALP, soil alkaline phosphatase activity. Blue lines indicate negative relationships, while red lines indicate positive relationships. The microbial diversities are represented by the Chao1 and Simpson indexes based on the rarified same sequencing depth. The width of arrows indicates the relevance of significant standardized path coefficients *(P < 0.05)*. ***P < 0.001, **P < 0.01

Redundancy analysis (RDA) revealed the relationship between soil microbial community diversity and soil biochemical properties (Fig. 6 A). The first principal component of RDA accounted for 46.52% of the total variation, and the second principal component accounted for 26.49% of the total variation. So, all variables could be well explained. The results showed that soil biochemical properties (pH, C/N ratio, and soil enzyme activity) and the relative abundances of *Acidobacteria* and *Proteobacteria* were closely in the first quadrant, indicating that the relative abundances of *Acidobacteria* and *Proteobacteria* were greatly impacted by soil biochemical properties. In the third quadrant, the longest arrow for soil available Cd concentration indicated that soil available Cd concentration had the greatest impact on soil microbial diversity. Soil available Cd had a large angle with soil biochemical properties (pH, C/N ratio, and soil enzyme activity) and the relative abundances of *Acidobacteria* and *Proteobacteria*, indicating that soil available Cd negatively impacted soil biochemical properties and the relative abundances of *Acidobacteria* and *Proteobacteria*. H3B and H2B treatments were also closely located in the third quadrant, indicating that the bacterial community structure in the H3B and H2B treatments were similar.

To determine the main factors responsible for the change of microbial community structure and available Cd concentration in Cd-contaminated soil, the direct and indirect effects of soil biochemical properties (soil enzyme and pH) and microbial diversity on soil available Cd were determined using structural equation model (SEM) (Fig. 6B). The results showed that soil urease and alkaline phosphatase activities had negative correlations with soil available Cd (β = -0.752 and β = -0.757, *P < 0.001*), indicating that soil available Cd could suppress soil urease and alkaline phosphatase activities. Soil available Cd had negative correlation with microbial diversity (β = -0.743, *P < 0.001*), indicating that exogenous Cd could decrease soil microbial diversity. However, urease activity had positive correlation with soil microbial diversity (β = -0.829 and β = -0.757, *P < 0.001*), indicating that soil urease activity could increase soil microbial diversity.

## Discussions

The effects of cotton straw biochar and compound *Bacillus* biofertilizer on soil biochemical properties was evaluated in this study, and the relationships between Cd and soil biochemical properties were also measured. Previous studies have shown that the bioavailability of Cd in the soil may increase when soil pH decreases; while the soil adsorption of Cd may increase when soil pH increases [28, 29]. In this study, soil pH and EC increased after the application of cotton straw biochar, which is consistent with the results of Bandara et al. [18]. The increase of soil pH may be due to the conversion of basic cations (such as Ca, Mg, K, and Na) in biochar into oxides, hydroxyl oxides, and carbonates (ash), which adhere to biochar during pyrolysis [24, 30]. The dissolution of the alkaline substances and the application of compound *Bacillus* biofertilizer could also increase soil pH. In this study, soil pH increased by 0.95 unit after the application of compound *Bacillus* biofertilizer, which is consistent with the results of Blaya et al. [31]. It may be due to the high pH of the compound *Bacillus* biofertilizer. Besides, biochar could accelerate the dissolution of most salts in the soil [18], resulting in the increase of soil electrical conductivity (EC) [32]. The increase of soil EC after the application of compound *Bacillus* biofertilizer may be due to the interaction of compound *Bacillus* biofertilizer with inorganic and organic ions in the soil [33]. Moreover, El-Kherbawy et al. [34] have showed that the concentration of available Cd in the soil with pH greater than 7.2 was lower than that in the acid soil, indicating that a high soil pH could positively impact Cd fixation, and soil pH could increase after the application of biochar [35]. In this study, the soil available Cd concentration decreased after the application of cotton straw biochar (*P < 0.05*). Cd ions precipitate with the alkaline ions in the soil, which reduces the soil available Cd [16]. The oxygen-containing functional groups of cotton straw biochar (carboxyl, carbonyl, and ester) (Table 3) induce Cd fixation, and absorb Cd on the surface through surface complexation [36]. *Bacillus* subtilis in compound *Bacillus* biofertilizer is a gram-positive, rod-shaped, and aerobic bacterium in the soil. Due to the different cell wall structures, *Bacillus* subtilis is more likely to bind with metals than gram-negative bacterium. Teichoic acid associated with the cell wall is unique to gram-positive cells, and its phosphate group is a key component of metal uptake [2, 37]. In this study, the soil available Cd concentration decreased by 74.34% (*P < 0.05*) after the application of compound *Bacillus* biofertilizer with *Bacillus* as the main component (Fig. 1). This is mainly because compound *Bacillus* biofertilizer is rich in a large number of microbes, which can reduce the available Cd concentration in the soil through the combination with microbial cell walls [38].

**Table 3:** Biochemical properties of biochar and soil

Property	Biochar	Soil
pH	9.50	7.76
Total nitrogen (g·kg^−1^)	0.89	0.46
Total P (g·kg^−1^)	2.54	0.82
Organic matter (g·kg^−1^)	625	14.73
Total K (g·kg^−1^)	8.62	246.83
Total Cd (mg·kg^−1^)	0.002	0.25
Available Cd	-	0.121
Total salinity (g·kg^−1^)	-	3.36
Carboxyl (mmol·g^−1^)	0.20	-
Lactone (mmol·g^−1^)	0.25	-
Phenolic hydroxyl (mmol·g^−1^)	0.21	-

Soil enzyme activity is an important biological indicator to evaluate soil quality, especially to the evaluation of soils contaminated by heavy metals [39]. The urease, alkaline phosphatase, and catalase are the most sensitive to heavy metals [2, 40]. Microorganisms secrete large amounts of urease. The decomposition of urease and the formation of bicarbonate, ammonium, and hydroxyl ions could increase the pH. All the urease-producing isolates could increase the pH of medium, which may greatly impact the bioavailability of soil heavy metals [2]. The reason for the decrease of soil enzyme activity after the application of exogenous Cd is that the molecular reaction between heavy metals and enzyme-substrate complexes or protein active groups denatures enzyme protein and reduces enzyme activity [41, 42]. Yang et al. [43] have shown that soil urease, alkaline phosphatase, and catalase activities could be increased after the application of cotton straw biochar (*P < 0.05*). In this study, soil urease, phosphatase, catalase, and sucrase activities increased by 16.55%, 15.51%, 31.33%, and 24.50%, respectively (*P < 0.05*), after the application of cotton straw biochar. It may be due to that the application of cotton straw biochar improves the soil biochemical properties, creating a good soil micro-environment for soil microbes’ growth and metabolism. Thereby, soil enzyme activities are increased [44, 45]. Compound *Bacillus* biofertilizer also increased soil enzyme activity in this study [46]. The increase in soil enzyme activities may be attributed to the increase in soil organic matter brought by the applications of compound *Bacillus* biofertilizer and cotton straw biochar. High concentration of soil organic carbon could stimulate soil microbial activities and the secretion of enzymes [46, 47]. In this study, soil alkaline phosphatase and urease activities had a negative correlation with soil available Cd concentration (*P < 0.01*) (Fig. 6), indicating that the applications of compound *Bacillus* biofertilizer and cotton straw biochar could increase soil enzyme activity, thus increasing the fertility and quality of Cd contaminated soil.

Heavy metal stress not only negatively impacts soil biochemical properties, but also causes changes in composition, activity and function of soil microbial communities [48]. In this study, the application of Cd reduced the relative abundance of *Chloroflexi*, and increased the relative abundance of *Proteobacteria* (Fig. 3 C), they are the core bacteria in control group (Fig. 4). It may be due to the difference in the absorption of heavy metals by soil microbes [49]. Besides, the soil carbon and nitrogen cycle were the main factor affecting soil microbial community [50]. The range of soil C/N ratio of 3.5 - 19.5 is the most beneficial for the growth and composition of soil microbes [51]. Cotton straw biochar and compound *Bacillus* biofertilizer are rich in organic matter and nutrients [25], which could provide energy for soil microbial activities, thereby increasing soil microbial abundance and biomass [36, 45, 51]. When cotton straw biochar and compound *Bacillus* biofertilizer are applied to the soil, the concentration of carbon and nitrogen required by the growth of soil microbes are greatly increased [52, 53]. In this study, the C/N ratio of soil was 16.41 - 18.66 after the applications of cotton straw biochar and compound *Bacillus* biofertilizer (Table 1), indicating that it was a favorable condition for microbial community. Besides, the application of cotton straw biochar increased soil microbial diversity (Table 2) and the relative abundance of *Acidobacteria* (Fig. 3), *Bacteroidetes* are the core bacteria in cotton straw biochar group (Fig. 4). Compared with the treatments without modifier, the application of cotton straw biochar obviously impacted the bacterial diversity and functions associated with soil carbon metabolism in Cd-contaminated soil, it may be due to the increase of soil C/N ratio caused by the high nutrient concentration of biochar [5, 54]. Moreover, the variations in soil microbial community structure may also be due to the reduction of soil available Cd concentration (Fig. 6). *Acidobacteria*, *Gemmatimonadetes*, *Actinobacteria* are core bacteria in compound *Bacillus* biofertilizer group (Fig. 4). Study have found that the number of soil bacteria increases from 18 to 9.8 × 10^7^ CFU·g^−1^ after the application of compound *Bacillus* biofertilizer [25], which is similar to the results of our study. In this study, the Cd ions in the soil were adsorbed and fixed by core bacteria after the applications of cotton straw biochar and compound *Bacillus* biofertilizer, thereby the Cd toxicity could be reduced and the microbial diversity could also be changed. The dominant phylum (*Proteobacteria* and *Cyanobacteria*) in the soil is related to specific soil enzyme. These microbes absorb heavy metal ions in the contaminated soil. Thus, the soil enzymes activity and bacterial metabolic functions could be increased (biosynthesis of amino acids and ribosome) (Fig. 5), and the soil quality could be improved [7, 55].

## Conclusions

In this study, the application of cotton straw biochar and compound *Bacillus* biofertilizer could improve soil pH, EC, C/N ratio, soil enzyme activity, as well as the relative abundance and metabolic function of *Acidobacteria*, *Gemmatimonadetes*, and *Bacillus*, while reduce the soil available Cd by 60.24% and 74.34%, respectively through adsorption. Besides, the key bacteria in cotton straw biochar (*Bacteroidetes*) and the *Bacillus* in compound *Bacillus* biofertilizer (*Acidobacteria*, *Gemmatimonadetes*, and *Actinobacteria*) also play a positive role in the immobilization of Cd. In general, compound *Bacillus* biofertilizer is better than cotton straw biochar in fixing soil Cd and improving soil environmental quality, which has great potential for the remediation of Cd-contaminated alkaline soils in arid and semi-arid areas.

## Methods

### Experimental site

This study was conducted at the Experimental Station of Agricultural College of Shihezi University, Xinjiang Province, China (44°18′42.37″N, 86°03′20.72″E), where there has a temperate arid continental climate. The average annual temperature is 7.5 - 8.2 ℃. The annual sunshine duration is 2318 - 2732 h, the frost-free period is 147 - 191 d, the annual rainfall is 180 - 270 mm, and the annual evaporation is 1000 - 1500 mm [27]. The soil texture is sandy loam.

### Preparation of experimental materials

Soils were collected from the cotton field with twenty-five years of continuous cropping in the study area. After removing residues, soils were air-dried and sieved through 2 cm and 0.19 mm sieves to determine soil physical and chemical properties (Table 3). Solid CdCl_2_·5H_2_O was mixed with the soil to prepare soil samples with different Cd concentrations. Solution (1.2 g·L^−1^of Cd^2+^) of 10 mL, 20 mL, and 40 mL were mixed with 12 kg soil to prepare the soil samples with 0.25 (H0), 1 (H1), 2 (H2), and 4 (H3) mg·kg^−1^ exogenous Cd^2+^. These levels were equivalent to three, six, and eleven times of the average soil Cd concentration globally [56, 57]. Finally, soil samples were stored for 60 d for subsequent tests [58].

Cotton straw biochar was prepared using anaerobic pyrolysis of cotton straw at 450 °C for 6 h, with a resultant biochar conversion rate of 37.5%. Cotton-straw biochar (B) was prepared using cotton stalk according to [59]. Cotton straw biochar was air-dried and sieved through a 0.2 mm sieve, and then the biochemical properties, including pH, organic matter, total nitrogen, total phosphorus, and total potassium, were measured [20]. Dried cotton straw biochar of 0.5 g was accurately weighed and digested with a mixture of nitric acid and muriatic acid (v:v=1:3) (Guaranteed reagent). The Cd concentration of cotton straw biochar was determined using the Hitachi Z2000 graphite atomic absorption spectrophotometer (PinAAcle900T, PerkinElmer, USA) (Table 3). The compound *Bacillus* biofertilizer (J) containing dominant functional bacteria of *Bacillus* was purchased from Shandong lvlong Biotechnology Co., Ltd, China, and the biochemical properties were measured according to the *Standards of Microbial Inoculants in Agriculture* (SMIA, National Standard of China, GB20287-2006). Compound *Bacillus* biofertilizer was sieved through a 0.2 mm sieve. The Colony-Forming Units (CFU) was greater than or equal to 20 billion·g^−1^, and the miscellaneous bacteria rate was less than 0.4%. The moisture was less than 10%, and pH was 7.8. Total Cd concentration was 0.0001 mg·L^−1^, total nitrogen concentration was 900 mg·L^−1^, and total organic carbon concentration was 3791 mg·L^−1^.

### Experimental design

The experiment employed a randomized block design with two factors. Four levels of soil Cd concentration were set, which were 0.25 (H0), 1 (H1), 2 (H2), and 4 (H3) mg·kg^−1^, and two modifiers were applied (T means no modifier). There were twelve treatments in total, and each treatment had five replicates (Table 4). Cd-contaminated soil (12 kg) was mixed with 3% (w/w) cotton straw biochar and 1.5% (w/w) compound *Bacillus* biofertilizer separately, and transferred into ceramic pots with a height of 40 cm and a diameter of 25 cm. After that, they were stored in a greenhouse (25 °C) for one week. Soils were irrigated with deionized water to keep the water holding capacity at 60%. Rhizosphere soil samples were collected after 120 days of cultivation. Part of the soil samples was air-dried for the analysis of soil pH, enzymes, total Cd concentration, and available Cd concentration; the other was sieved through a 2 mm sieve and stored at -80 °C for microbial diversity analysis.

**Table 4:** Amount of Cd, biochar, and biofertilizer in each treatment

Treatments	Cd (mg·kg^−1^)	Biochar (%)	Biofertilizer (%)
H0T	0.25	0	0
H0B	0.25	3%	0
H0J	0.25	0	1.5%
H1T	1	0	0
H1B	1	3%	0
H1J	1	0	1.5%
H2T	2	0	0
H2B	2	3%	0
H2J	2	0	1.5%
H3T	4	0	0
H3B	4	3%	0
H3J	4	0	1.5%

T, no modifiers; B, 3% biochar was applied; J, 1.5% biofertilizer was applied; H0, no Cd; H1, 1 mg·kg^−1^ of Cd was applied; H2, 2 mg·kg^−1^ of Cd was applied; H3, 4 mg·kg^−1^ of Cd was applied

### Determination of soil indices

#### Soil biochemical indices

Soil pH was measured with a pH meter (Thermo Orion 920 A, Thermo Orion, USA) (soil: water = 1: 5). Soil organic carbon was measured with the wet oxidation method [60]. Soil total nitrogen concentration was measured with a semi-micro-Kjeldahl procedure [18]. Soil available Cd concentration was measured with the diethylenetriaminepentaacetic acid (DTPA) extraction method using a graphite furnace atomic absorption spectrophotometer (Z2000, Hitachi, Tokyo, Japan) [18]. Soil urease activity was measured with indophenol-blue colorimetry, invertase activity was measured using 3,5-dinitrosalicylic acid colorimetry, alkaline phosphatase activity of disodium phenyl phosphate was measured using colorimetric method, and soil catalase activity was determined using the volumetric method [18].

To determine the water holding capacity, damp soil of 50 g was accurately weighed and transferred into the tube with mesh base (3.5 cm in diameter and 5 cm in length). Then, the tube was placed in a container with water and allowed to be wetted by capillary action. When the soil surface became glossy, soil cores were removed from the water and allowed to drain until they stopped dripping. The soil in the cores was then gently removed and weighed. The water holding capacity of the soil was determined as the weight of water held in the soil cores compared with the oven-dry weight (105 °C) of the sample [61, 62].

### Analyses of the structure and diversity of soil microbial community

DeoxyriboNucleic Acid (DNA) was extracted from soil samples using the E.Z.N.A.® Soil DNA Kit (OMEGA, USA). Soil samples stored at -80 °C were weighed to extract the total DNAs according to the instructions of the kit. After that, the DNAs were stored at -80 ℃. Polymerase Chain Reaction (PCR) amplification was conducted using 0.8 µL of bacterial synthetic primers (Forward Primer: ACTCCTACGGGAGGCAGCAG; and Reverse Primer: GGACTACHVGGGTWTCTAAT). 16 S rRNA gene V3-V4 was targeted using the primer set. The product was cycled 30 times at 95 °C. The PCR products were detected using 2% agarose gel electrophoresis, and then AxyPrep DNA Gel Extraction Kit and Quantus ™ Fluorometer were used to purify and quantify the products [63]. Illumina MiSeq System (Milq PE300 platform of Illumina compan, USA) was used for sequencing by Shanghai Meiji Technologies Corporation, China.

### Data process and analysis

Data of soil available Cd and pH were subjected to regression analysis using a Duncan test at *P < 0.05* (SPSS 18.0). Redundancy analysis (RDA) was used to evaluate the effect of soil biochemical variables on the microbial community composition based on a Bray-Curtis distance matrix. The Pearson’s correlation test was used to examine the correlation between the relative abundance of microbes and environmental factors (soil available Cd, soil pH, and soil enzyme). R software (version 3.6.1) and Origin 8.0 software (Origin Lab, Massachusetts, USA) were used for plotting.

Sequences were clustered at a 97% similarity level using Quantitative Insights Into Microbial Ecology (QIIME) package (version 1.9.1), and operational taxonomic units (OTU) were obtained, with 0.005% as threshold. To compare the species richness of soil bacteria after applying cotton straw biochar and compound *Bacillus* biofertilizer, the total community richness was calculated using different statistical methods, including Chao1, Simpson, and Coverage indices. Phylogenetic Investigation of Communities by Reconstruction of Unobserved States (PICRUSt2) software (https://github.com/picrust/picrust2/wiki) predicted the functions (MetaCyc database) of soil bacteria. The structural equation model (SEM) analysis was performed using AMOS 20.0 software (AMOS, IBM, USA) with a maximum-likelihood method [35].

## Data Availability

The datasets used and/or analysed during the current study available from the corresponding author on reasonable request.
